# Prevention of aspiration of nasopharyngeal airway

**DOI:** 10.4103/0019-5049.60511

**Published:** 2010

**Authors:** Paul J Grube, Dapeng Fan, Vijayasimha R Pothula, Charles T Vonfrolio, David Tsang, David Hoffman

**Affiliations:** Staten Island University Hospital, New York, USA; 1 Kings County Hospital, New York, USA

**Figure 1:**
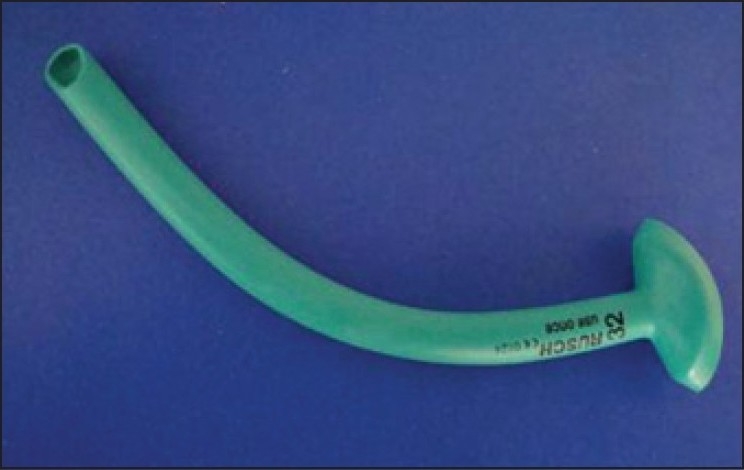
Nasopharyngeal airway

Sir,

Nasopharyngeal airway (NPA) [Figure 1] is an airway accessory used to prevent upper airway obstruction by tongue and helps in nasotracheal suctioning. It is the least invasive method of safely managing upper airway obstruction. There are several cases of nasopharyngeal airway aspiration reported in literature.[1–3]

Although rare, NPA aspiration can occur. Several factors contribute to NPA aspiration. First, the NPA model can be very supple, lubricated and with a particularly thin flexible flange that is unable to stop distal migration once started. Second, the patient may have rubbed his/her nose briefly prior to the incident. This may initiate the event possibly combined with a more “nasal” breathing from maxillary occlusion to further advance airway. Finally, perhaps the nasal passage itself may have dilated somewhat from the more rigid nasotracheal tube used during surgery, thus facilitating passage of the airway that was of appropriate diameter and length.

NPA aspiration can be prevented by use of barriers to distal migration, safety pins and endotracheal tube connectors; placement of NPAs in the nasal passage opposite that used for intubation may also be of value. NPA aspiration can be prevented by encircling 1-cm wide elastopore tape at the proximal end of the nasopharyngeal airway and securing it to the external nose in the same way as a nasotracheal tube is secured[3–5]
